# Rheological Properties of Cement Paste with Nano-Fe_3_O_4_ under Magnetic Field: Flow Curve and Nanoparticle Agglomeration

**DOI:** 10.3390/ma13225164

**Published:** 2020-11-16

**Authors:** Dengwu Jiao, Karel Lesage, Mert Yucel Yardimci, Khadija El Cheikh, Caijun Shi, Geert De Schutter

**Affiliations:** 1 Magnel-Vandepitte Laboratory, Department of Structural Engineering and Building Materials, Ghent University, 9052 Ghent, Belgium; Dengwu.Jiao@UGent.be (D.J.); Karel.Lesage@ugent.be (K.L.); MertYucel.Yardimci@UGent.be (M.Y.Y.); khadija.el.cheikh@bbri.be (K.E.C.); 2 Key Laboratory for Green and Advanced Civil Engineering Materials and Application Technology of Hunan Province, College of Civil Engineering, Hunan University, Changsha 410082, China; cshi@hnu.edu.cn

**Keywords:** cement paste, rheology, nano-Fe_3_O_4_, magnetic field, parallel plate

## Abstract

Understanding the influence of magnetic fields on the rheological behavior of flowing cement paste is of great importance to achieve active rheology control during concrete pumping. In this study, the rheological properties of cementitious paste with water-to-cement (w/c) ratio of 0.4 and nano-Fe_3_O_4_ content of 3% are first measured under magnetic field. Experimental results show that the shear stress of the cementitious paste under an external magnetic field of 0.5 T is lower than that obtained without magnetic field. After the rheological test, obvious nanoparticle agglomeration and bleeding are observed on the interface between the cementitious paste and the upper rotating plate, and results indicate that this behavior is induced by the high magnetic field strength and high-rate shearing. Subsequently, the hypothesis about the underlying mechanisms of nanoparticles migration in cementitious paste is illustrated. The distribution of the nanoparticles in the cementitious paste between parallel plates is examined by the magnetic properties of the powder as determined by a vibrating sample magnetometer. It is revealed that the magnetization of cementitious powders at different sections and layers provides a solid verification of the hypothesis.

## 1. Introduction

Rheology control is essential in striking a balance between contradicting property requirements in different casting processes for the same concrete mixture, e.g., high flowability and low thixotropic structural build-up in pumping processes against high structuration rate in formwork casting [1,2]. Currently available rheology control in cement-based materials is generally achieved by pre-adding mineral additives or chemical admixtures according to the relationship between the rheological properties and mixture proportions [3,4,5]. Once the cement-based material is mixed, however, no further intervention is possible to adjust the properties. In other words, post-mixing control of fresh concrete properties cannot be achieved by currently available property control methods.

In this case, an innovative casting concept “SmartCast” has been proposed [6,7,8], aiming at active rheology control (ARC) and active stiffening control (ASC) of cement-based materials. By activating an external trigger signal, the structuration rate and rheological properties of cementitious materials containing responsive additives could be artificially controlled on-demand. This contributes to more reliable and smarter pumping and casting operations. One potential approach to achieve the active control is by adding magnetic particles to the mixture in combination with exploiting an external magnetic field. The theoretical foundations are the concept of magnetorheological (MR) fluid, which transitions from Newtonian fluid to semi-solid state immediately after applying an external magnetic field [9], resulting in a significant increase in yield stress and plastic viscosity. After removing the external magnetic field, however, the MR fluid reversibly changes from semi-solid to liquid. Therefore, controllable MR fluid by applying external magnetic field is widely used in many engineering applications [10,11]. With regard to cementitious materials, Nair and Ferron [12,13] found that the rheological properties (i.e., storage modulus and static yield stress) of fresh cement paste containing carbonyl iron particles could be significantly altered by applying an external magnetic field. In our previous studies [14,15], the early structural build-up of cementitious paste containing nano-Fe_3_O_4_ particles under various magnetic fields was comprehensively investigated, based on the relationship between storage modulus, loss modulus, and phase angle.

Rheological properties, with yield stress and plastic viscosity as two intrinsic physical parameters [16], are of great importance in describing flowability and workability, evaluating pumpability, and predicting formwork filling of cement-based materials. Wallevik et al. [17,18] stated that the rheograph of yield stress against plastic viscosity can be used to optimize the rheological properties of fresh concrete. Kwon et al. [19] illustrated that the rheological properties of the lubrication layer can be used to predict the flow rate of pumped concrete. Roussel et at. [19,20] pointed out that the numerical simulation of non-Newtonian fluids considering various rheological parameters was a valuable approach to determine the minimum flowability guaranteeing adequate formwork filling and acceptable stability. Moreover, the rheological properties can be used to optimize the flowability of 3D printing concrete, achieving a balance between pumpability, extrudability, and buildability [21,22]. Accordingly, active control of rheological properties is beneficial to advancing the casting technique of fresh concrete. In the case of MR fluids under an external magnetic field, the plastic viscosity can increase about 10^5^–10^6^ times and the field-induced yield stress increases to 100 kPa [9]. Furthermore, the shear stress of MR fluids generally shows a plateau behavior at relatively low shear rates, while at higher shear rates, the shear stress gradually increases with increasing shear rate [23,24,25] due to the equilibrium between the structural breakdown and the build-up of chain structures. For the flowing cementitious materials containing magnetic nanoparticles, however, the rheological behavior under external magnetic field is still not fully understood.

In the present study, the rheological properties of cementitious paste containing nano-Fe_3_O_4_ particles in the presence of an external magnetic field are first experimentally investigated. Nanoparticle agglomeration and the bleeding phenomena are observed after the rheological tests. Subsequently, possible influencing factors on nanoparticles migration in cement paste between the parallel plates of the rheometer are examined. The hypothesis about the underlying mechanisms of migration of the nanoparticles in cement paste under the synergistic effect of an external magnetic field and shearing is verified by analyzing the magnetic properties of powders using a vibrating sample magnetometer. This study contributes to a fundamental understanding of the magneto-rheological responses of flowing cementitious paste with magnetic nanoparticles.

## 2. Materials and Methods

**Figure 1:**
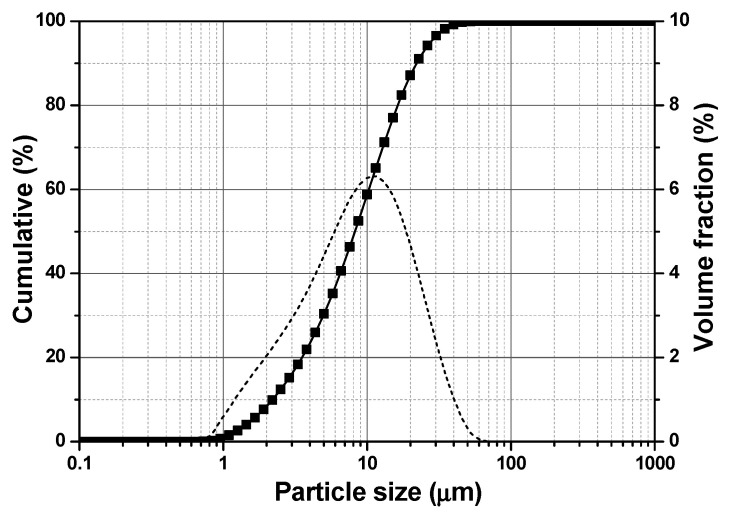
Particle size distribution of the Portland cement.

**Figure 2:**
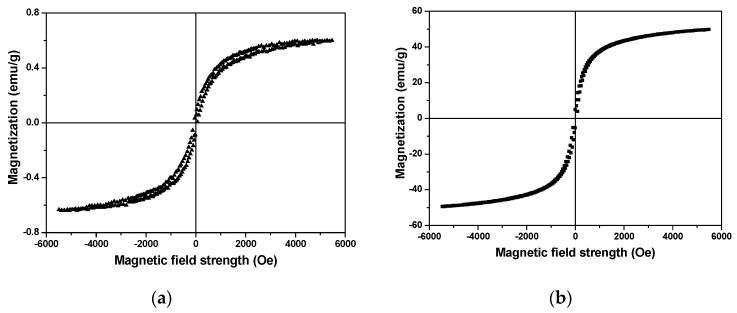
Magnetization versus magnetic field strength curve of (**a**) Portland cement and (**b**) nano-Fe_3_O_4_ particles.

Ordinary Portland cement (OPC) CEM I 42.5 N and spherical iron oxide nano-Fe_3_O_4_ particles (MNPs) with Fe_3_O_4_ purity higher than 98% (from US Research Nanomaterials, Inc, Houston, TX, USA) were used. The chemical composition and particle size distribution of the cement are shown in Table 1 and Figure 1, respectively. The apparent density and median particle size (D50) of the cement particles are 3.15 g/cm^3^ and 9.459 μm, respectively. According to the manufacturer, the particle size and apparent density of the nano-Fe_3_O_4_ particles are 20–30 nm and 4.95 g/cm^3^, respectively. The curves of magnetization versus magnetic field strength of the cement and nano-Fe_3_O_4_ particles obtained from a vibrating-sample magnetometer (VSM) (VSM-550, Dexing Magnet, Xiamen, China) are presented in Figure 2. A commercial polycarboxylate ether superplasticizer (PCE) (MasterGlenium 51, BASF Nederland B.V., Oosterhout, Netherlands) was employed. All samples were prepared using de-ionized water.

A cementitious paste with water-to-cement (w/c) mass ratio of 0.4 and MNP content of 3% (by mass of total cement paste, i.e., cement + water) was prepared, named 0.4_3%MNPs. The paste shows excellent stability and appropriate flowability after mixing. Pure cement paste with a w/c ratio of 0.4 was also prepared. All the cementitious pastes were mixed using a rotational rheometer equipped with a home-made helix geometry, providing a repeatable initial state of paste samples. The details of the geometric parameters of the helix geometry and the mixing procedure can be found in [14].

**Figure 3:**
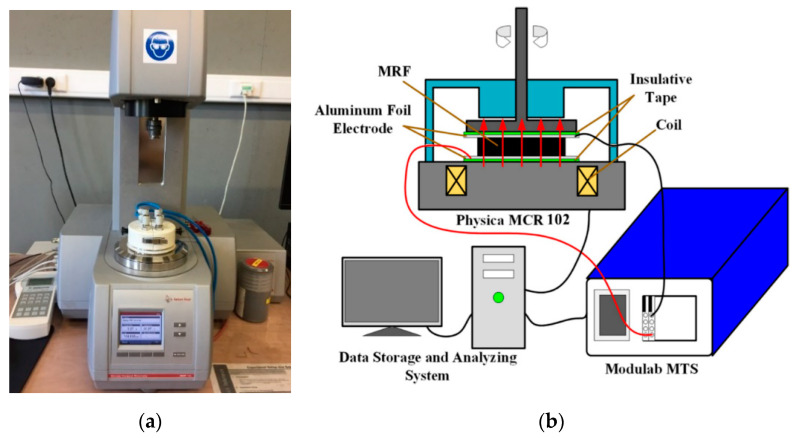
(**a**) MCR 102 rotational parallel plate rheometer equipped with a magnetic cell and (**b**) the corresponding schematic diagram.

**Figure 4:**
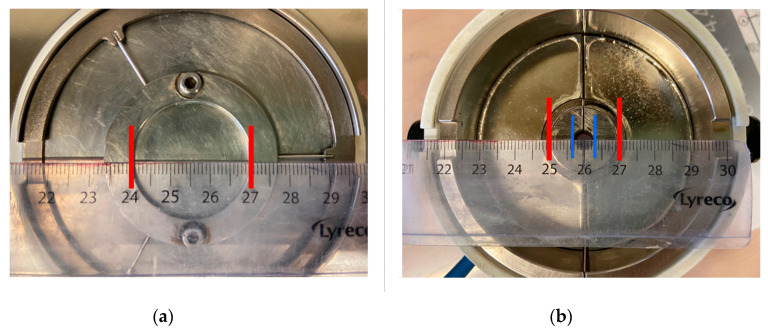
Geometrical parameters of (**a**) the bottom plate of the rheometer and (**b**) the upper yoke of the magneto-rheological device.

**Figure 5:**
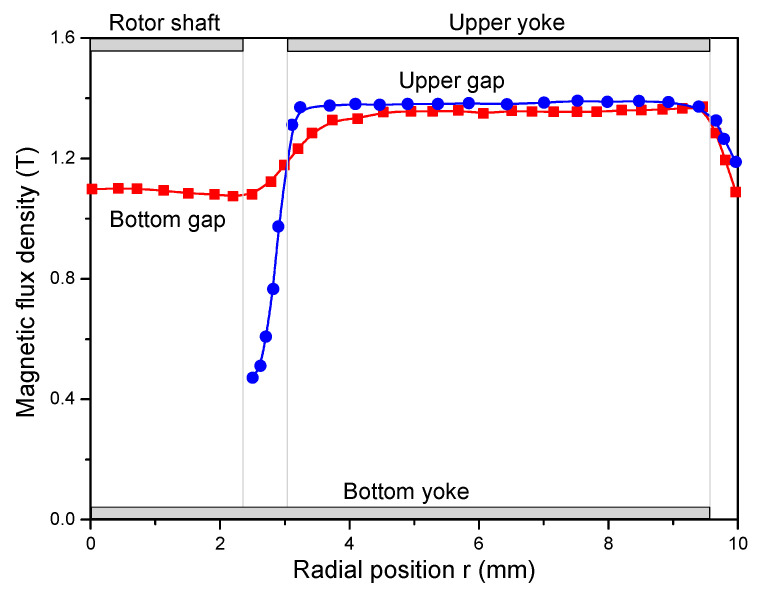
Simulated flux density profiles in the middle of the shear gaps filled by magnetorheological (MR) fluid at an input current of 3 A and gap of 1.1 mm [26].

The flow curve of the cementitious pastes was evaluated using a rotational parallel plate rheometer (MCR 102, Anton Paar, Graz, Austria) equipped with a magneto-rheological device (Physica MRD 170 + H-PTD200), as shown in Figure 3. The geometrical parameters of the lower plate and the upper yoke of the magneto-rheological device are presented in Figure 4. The diameters of the bottom plate and the upper plate are 30 mm and 20 mm, respectively, and therefore, the effective diameter of the parallel plates is 20 mm. The simulated radial flux density profiles with the middle of the parallel gaps filled with MR fluid are presented in Figure 5. It can be seen that, except for the center and the periphery of the plate, a comparatively homogeneous magnetic field perpendicular to the plates can be applied to the sample by controlling the current of the electromagnetic coils [26]. In the present research, the gap between the upper and lower plates was fixed at 1 mm.

**Figure 6:**
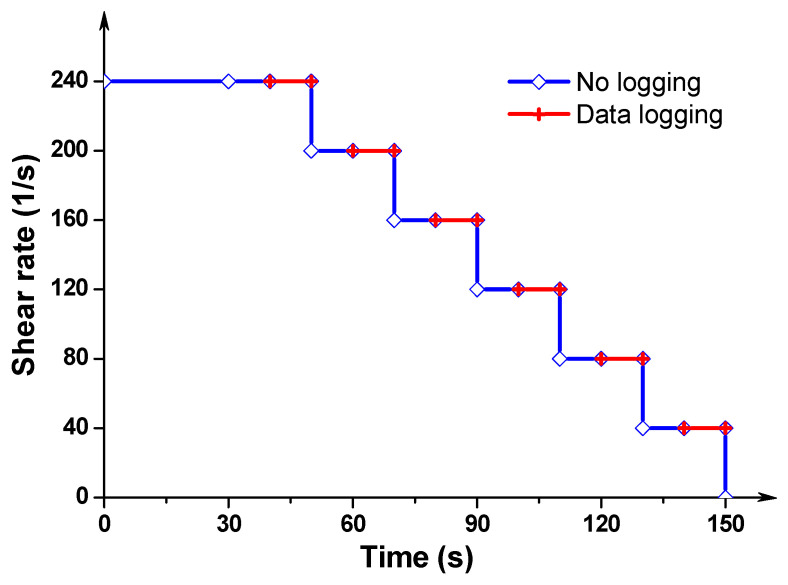
Testing protocol of shear curves.

After pouring the sample into the plate, a pre-shear procedure with shear rate of 100 s^−1^ was first applied for 30 s, and, then the sample was magnetized under an external magnetic field (0 T or 0.5 T) for 5 min. Afterwards, the sample was sheared at shear rate of 240 s^−1^ for 30 s to obtain a homogeneous state. The flow curve test protocol consists of decreasing the shear rate from 240 s^−1^ to 40 s^−1^ by six steps (i.e., 240 s^−1^, 200 s^−1^, 160 s^−1^, 120 s^−1^, 80 s^−1^ and 40 s^−1^), as presented in Figure 6. Each rotational shearing step lasted for 20 s, and only the last 10 s were used to record the data. The resulting six points of shear rate versus shear stress were fitted using the Bingham model (see Equation (1)) to calculate the rheological parameters.

(1) $$\tau =\tau_{0}+\mu \dot{\gamma }$$

where *τ* and *τ*_0_ are the shear stress and yield stress, respectively; *μ* is the plastic viscosity and $\dot{\gamma }$ is the shear rate. During the magnetization process and rheological tests, the temperature was controlled at 20 ± 0.5 °C. For each mix proportion, the rheological tests were repeated at least three times using fresh samples. The error bar in the following figures is the standard deviation of three repeatable results.

## 3. Results

**Figure 7:**
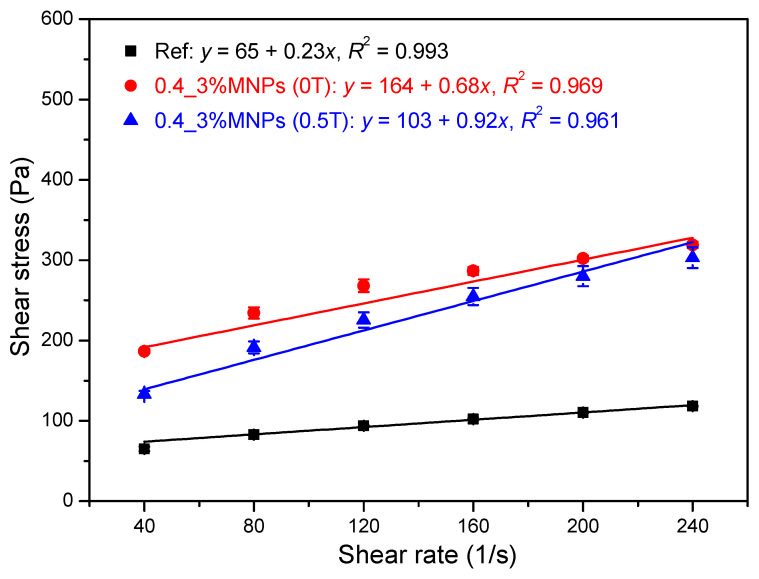
Effect of magnetic field on the flow curve of cementitious pastes.

**Figure 8:**
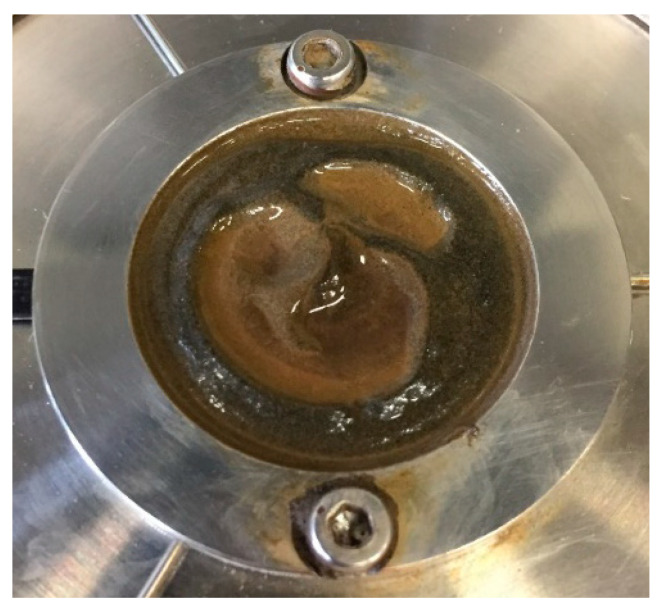
Upper surface of the cementitious paste (0.4_3%MNPs) after rheological tests.

The effect of magnetic field on the flow curve of the cementitious paste (0.4_3%MNPs) is shown in Figure 7. The flow curve of pure cement paste with a w/c ratio of 0.4 (Ref) is also presented as a comparison. As expected, without an external magnetic field, the addition of nano-Fe_3_O_4_ particles increased the measured shear stress, yield stress, and plastic viscosity due to the high water demand of the nanoparticles as well as the increase in friction and collision between solid particles. Besides, the possible formation of small agglomerates of nanoparticles is also responsible for the increase in the rheological properties. After applying an external magnetic field of 0.5 T, the measured shear stresses were lower than that obtained without a magnetic field, especially at relatively low shear rates. As a result, comparing with the values obtained without magnetic field, lower yield stress and higher plastic viscosity were achieved in the presence of the external magnetic field of 0.5 T. This is totally different from the results of typical MR fluids [9,27,28], which show that dynamic yield stress significantly increases under an external magnetic field. In this study, this abnormal phenomenon can be explained by the distribution of nanoparticles in the cementitious suspensions during the rheological test. The surface of the cementitious paste after the rheological tests was checked, as shown in Figure 8. Apparent agglomeration and/or migration of nanoparticles as well as bleeding on the interface between the cementitious paste and the rotating upper plate can be observed. These phenomena possibly appeared after high-rate shearing during the flow curve test under the magnetic field. The agglomeration of nanoparticles and bleeding result in the cementitious paste having weaker structural strength [29,30], exhibiting low resistance to the applied shearing. Consequently, a high slope of the flow curve was obtained due to slippage, and thus, lower yield stress and higher plastic viscosity were observed compared to the cementitious paste with dispersed nanoparticles. In the following sections, the possible influencing factors and underlying mechanisms for the nanoparticle agglomeration and/or migration are examined and discussed.

## 4. Discussion: Agglomeration/Migration of Nanoparticles

### 4.1. Influencing Factors

**Figure 9:**
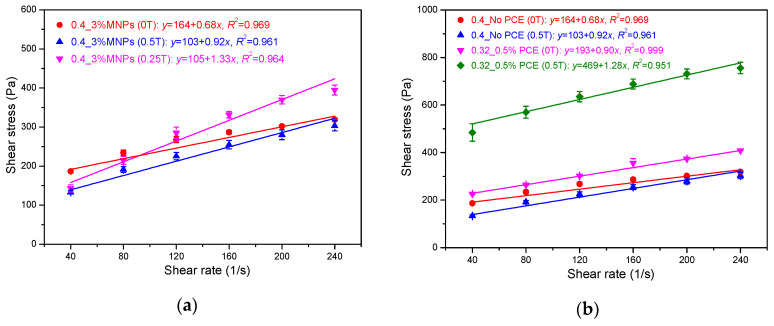
Effect of (**a**) various magnetic fields and (**b**) paste mediums on the flow curve.

**Figure 10:**
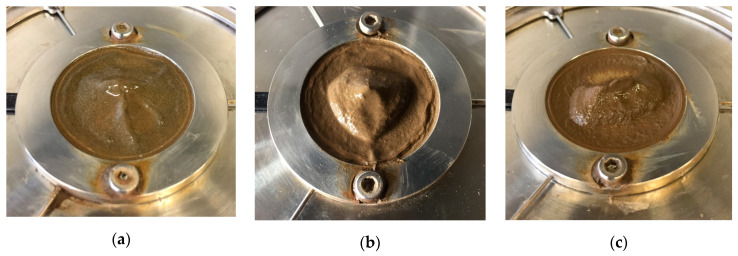
Interfaces between cementitious paste and upper rotating plate after rheological tests. (**a**) 0.4_3%MNPs (0.25T)_Shearing, (**b**) 0.4_3%MNPs (0.5T)_No shearing, and (**c**) 0.32_0.5%PCE_3%MNPs (0.5T)_Shearing.

The agglomeration and migration of nano-Fe_3_O_4_ particles in the cementitious paste (0.4_3%MNPs) might be attributed to the coupled effect of high magnetic field strength and high-rate shearing, as no bleeding was observed for the cementitious paste after rheological tests without magnetic field. The rheological properties of the cementitious paste under an external magnetic field of 0.25 T were further evaluated, as depicted in Figure 9a. Higher measured shear stresses were obtained compared to the values obtained under 0.5 T magnetic field. The surface of the cementitious paste was checked after performing the rheological testing procedure, as shown in Figure 10a. It can be observed that, for the same mixture, the extent of bleeding and nanoparticles agglomeration under the magnetic field of 0.25 T was less pronounced compared to that under the magnetic field of 0.5 T. This can be attributed to the fact that the magnetic field with 0.25 T is insufficient to facilitate migration of the formed magnetic clusters of nano-Fe_3_O_4_ particles in the cementitious suspension. In an additional experiment, the cementitious paste with a w/c ratio of 0.4 and 3 wt.% nano-Fe_3_O_4_ was magnetized under an external magnetic field of 0.5 T for 10 min without shearing. After removal of the upper plate, the surface of the cementitious paste was checked, as shown in Figure 10b. It can be seen that the cementitious paste was homogeneous after magnetization (without shearing) for 10 min. This, in turn, indicates that the shearing facilitates the accumulation of excessive water and magnetic nanoparticles on the upper surface of the cementitious paste. Remarkably, when the magnetic field direction changed from top to bottom, the nanoparticles still agglomerated on the interface between the cementitious paste and the upper rotating plate, and there was no nanoparticle agglomeration observed on the bottom plate.

Furthermore, a cementitious paste with a w/c ratio of 0.32, PCE of 0.5%, and nano-Fe_3_O_4_ of 3 wt.% (0.32_0.5%PCE_3%MNPs) was also prepared to undergo the same rheological procedure (i.e., magnetization and shearing) under an external magnetic field of 0.5 T. The shear curve results are presented in Figure 9b. In the absence of magnetic field, the yield stress and plastic viscosity of the cementitious paste were 193 Pa and 0.90 Pa·s, respectively. In the presence of a magnetic field of 0.5 T, however, the yield stress and plastic viscosity increased up to 469 Pa and 1.28 Pa·s, respectively. The surface of the paste after removing the upper plate was shown in Figure 10c. No bleeding or agglomeration of magnetic nanoparticles was observed for the cementitious paste with low water content and superplasticizer. This can be explained by the relatively high rheological properties of the cementitious suspension, which exert a significant resistance to the migration of nanoparticles under the coupled effects of magnetic field and rotational shearing.

Overall, under an external magnetic field of 0.5 T, the bleeding and nanoparticles agglomeration of cementitious paste with a w/c ratio of 0.4 and nano-Fe_3_O_4_ particles of 3 wt.% are the result of the combination of high magnetic field and rotational shearing.

### 4.2. Underlying Mechanisms: Hypothesis

**Figure 11:**
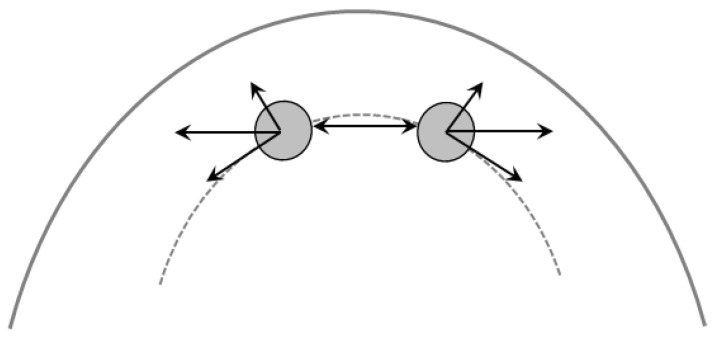
Schematic diagram illustrating the interactions between two particles in a curved geometry (based on [32]).

In the available literature, two main mechanisms are widely used to explain particle migration of poly-dispersed concentrated suspensions in parallel plates, i.e., shear-induced migration [31] and curvature-induced migration [32]. According to the shear-induced migration theory, hydrodynamic interactions between solid particles depend on the gradients of shear rate and concentration. For an initially inhomogeneous suspension, particles in the high shear rate (or concentration) zone experience a larger rate of interactions compared to the region of low shear rates (or concentration), resulting in particles migrating and diffusing down with shear rate (or concentration) gradient. The larger the particle size is, the greater the effect of shear-induced migration is. In the case of poly-dispersed suspensions, coarser particles have a tendency to migrate from the outer side to accumulate in the center of the plate. The mechanism of the curvature-induced migration can be explained by the schematic diagram presented in Figure 11. Assuming two equally sized particles colliding each other under shear flow, the interaction force can be divided into two components: one is the direction radially outward, and the other is the direction tangential to the local streamwise direction. After collision, the two particles are separated and tend to migrate outward to the region with relatively low curvature. For bi-dispersed suspensions, larger particles tend to migrate radially outward under the effect of curvature-induced migration. For the mono-dispersed suspensions in a parallel-plate geometry, the shear-induced effect and the curvature-induced migration counteract each other, and thus, there is no significant variation in the radial concentration profile [33,34].

In the current study, the nanoparticle agglomeration after rheological tests under a magnetic field can be possibly explained by the combination of magnetic force and shear-induced migration. The nano-Fe_3_O_4_ particles accumulate to magnetic clusters in the cementitious suspensions after magnetization for 5 min. The size of some formed clusters is even larger than that of cement particles [35]. During the flow curve test, the upper plate rotates and the lower plate remains stationary. The flow of the sample between the plates can be regarded as a steady laminar flow. Since the interaction between magnetic clusters is stronger than that between individual particles [27], although some chains or clusters are broken down during shearing, new clusters are formed simultaneously under the external magnetic field. Furthermore, the movement of particles provides extra possibilities for the magnetic nanoparticles to contact each other, which is beneficial in forming larger magnetic clusters.

**Figure 12:**
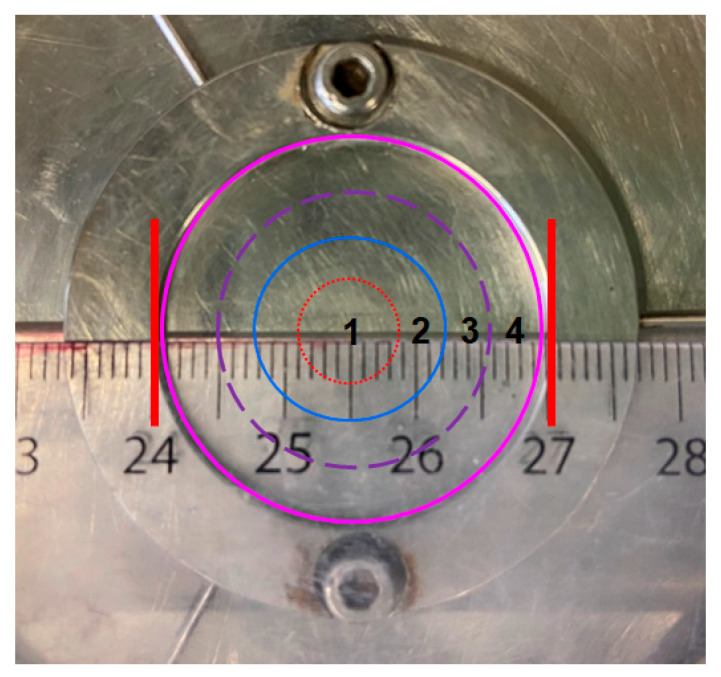
Four divided sections of the lower plate.

When dividing the lower parallel plate into four sections (S1, S2, S3 and S4), as shown in Figure 12, the order of the curvature in the four sections is S1 > S2 > S3 > S4. During the rheological shearing test, the shear rate (stress) linearly increases from the center to the periphery of the plate, indicating the following order of shear rate: S1 < S2 < S3 < S4. According to the simulation results in Figure 5, it can be seen that S2 and S3 have similar magnetic flux densities whereas S1 and S4 have relatively low magnetic flux densities. Consequently, a slight gradient of magnetic flux density exists when considering the whole parallel plate.

Without an external magnetic field, the nanoparticles are randomly distributed in the cement paste. Under the short rotational shearing, the particles migration is limited due to the competition between shear-induced and curvature-induced effects. This is also presumed to be applicable to the case with low strength magnetic field. Consequently, the behavior of nanoparticle agglomeration is not observed after rheological shearing test without a magnetic field and under a magnetic field of 0.25 T. In the presence of an external magnetic field of 0.5 T, the magnetic nanoparticles have a strong tendency to migrate from a low flux density region to a high flux region, i.e., from the periphery (S4) and the center (S1) of the plate to the intermediate sections (S2 and S3). Without additional shear stress, i.e., at rest state, the potential migration of nanoparticles under the effect of a magnetic force gradient is restricted by the viscous cementitious suspension with high yield stress. After applying an external rotational shearing, however, the resistance exposed to the magnetic nanoparticles by the suspension medium significantly decreases, as reflected by the fact that dynamic yield stress is generally lower than static yield stress. In the low-curvature section, e.g., S4, the inward migration effect induced by the shear rate gradient and magnetic force gradient is supposed to be stronger than the curvature-induced outward migration effect, resulting in larger magnetic clusters of nanoparticles gradually migrating from the outer sections to the inner sections. By contrast, in the high-curvature section, e.g., S1, the coupled effect of magnetic force and curvature-induced migration dominates compared with the shear-induced effect, leading to nanoparticles migrating away from S1. Therefore, under the combination of shearing and magnetic field, the magnetic clusters migrate on the interface between cement paste and rotating plate, which contributes to the formation of nanoparticle agglomeration.

### 4.3. Experimental Verification: VSM Tests

**Figure 13:**
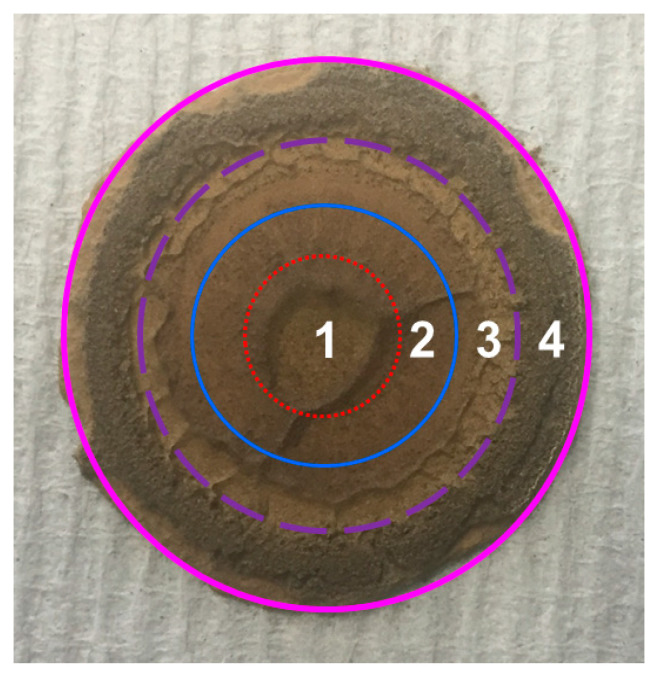
Sample preparation of paste powder by horizontally dividing four sections.

The abovementioned hypothesis is tentatively verified by VSM measurements. The VSM results reveal the magnetic properties of the tested cementitious powder, which could indirectly identify the concentration of nano-Fe_3_O_4_ particles of the paste at different sections. Higher saturation magnetization of the powder indicates higher concentration of magnetic nanoparticles [36]. A cementitious paste (0.4_3%MNPs) disc with thickness of 1 mm was prepared after rheological tests (including the magnetization for 5 min) under a magnetic field of 0.5 T. After finishing the rheological test, the magnetic field was removed. To eliminate the pull-off behavior of the upper plate, it was removed only after three h, so that the cementitious paste can keep its shape. Afterwards, the paste disc was kept in the plate of the rheometer until 24 h (without magnetic field), and then, the paste disc was hand-cut into four sections in the horizontal direction, as shown in Figure 13. Afterwards, all the small pieces of the cementitious paste were ground into powder, on which the VSM test was conducted at room temperature with magnetic field strength varying from −10,000 Oe to 10,000 Oe. The mass of ground powder of each section was around 50 mg. It should be mentioned here that the uneven surface of the same samples as visible in Figure 8, Figure 9, Figure 10, Figure 11, Figure 12 and Figure 13 is possibly attributed to the pull-off action of the upper plate.

**Figure 14:**
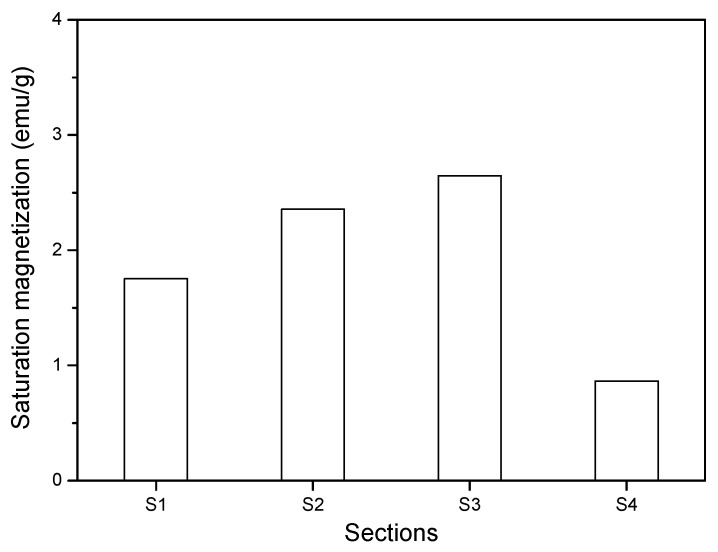
Saturation magnetization of the four sections after rheological tests.

The saturation magnetization of the ground powders obtained from different sections of the sample disc in Figure 13 is plotted in Figure 14. It can be seen that the saturation magnetization of the powder in S4 was much lower than that in other sections, indicating that the paste disc in S4 contains the lowest concentration of nano-Fe_3_O_4_ particles. This can be due to the low magnetic flux density, low curvature, and high shear rate in S4. The saturation magnetization in S3 was larger than that in S1 and S2. Despite the hypothesis assuming that magnetic clusters should migrate radially inward from the periphery to the inner sections of the plate, a relatively high number of nanoparticles in S3 compared to S2 can be attributed to the fact that an absolutely large amount of nanoparticles migrates away from S4. Another possible reason is that the short period of high-rate shearing is not sufficient for the nanoparticles and clusters to migrate to inner sections. Moreover, the saturation magnetization in S1 was slightly lower than that in S2 probably due to the adverse magnetic force gradient and curvature gradient playing more significant roles than the shear rate gradient, resulting in the nanoparticles adversely migrating from S1 to S2.

**Figure 15:**
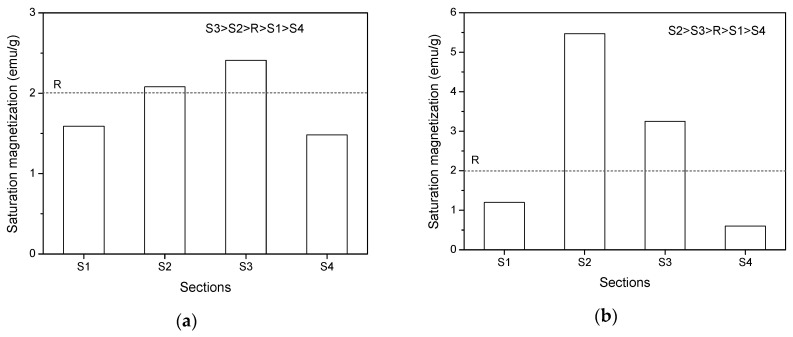
Saturation magnetization of cementitious powders in parallel plate after constant shearing at (**a**) 40 s^−1^ and (**b**) 240 s^−1^.

As aforementioned, under the combination of magnetic field and shearing, a large number of nanoparticles migrated from S4 to S3 and the short period of high-rate shearing resulted in a higher nanoparticles concentration in S3. To verify this statement, VSM measurement of the cementitious paste powders in the parallel plate with a gap of 1 mm after experiencing constant shearing with different rates (in the presence of 0.5 T magnetic field) was performed. Figure 15a,b shows the saturation magnetization of cementitious powders in the four sections after exposure to a constant shear rate of 40 s^−1^ (low-rate) and 240 s^−1^ (high-rate) for 120 s, which is similar to the duration of the rheological test. A reference cementitious paste with a w/c ratio of 0.4 and MNPs of 3% without experiencing any shearing test and magnetic field was also prepared, and the saturation magnetization of the powder obtained from this paste was determined by VSM as a comparison, as presented in the dash line (R) in Figure 15. As expected, the saturation magnetization in S3 was the highest after experiencing the low-rate shearing due to insufficient shearing. By contrast, the high-rate shearing action with the same duration resulted in a significant high-saturation magnetization in S2. This indicates that most of the magnetic nano-Fe_3_O_4_ particles accumulated in S2 after experiencing a sufficiently long period of high-rate shearing. The saturation magnetization values in S2 and S3 after high-rate shearing were higher than that in the same sections after low-rate shearing, whereas the magnetization values in S1 and S4 showed opposite behavior. This can be attributed to the facilitation effect of high-rate shearing to the migration of nanoparticles. Interestingly, the saturation magnetization of the reference cement paste was lower than that in S2 and S3 but higher than that in S1 and S4, regardless of the shear rate. This supports the nanoparticle migration from S4 to the inner zones as well as the adverse migration of nanoparticles from S1 to S2. The VSM experimental results provide a solid argument for the nanoparticle distribution in the parallel plates under the combination effect of magnetic field and rotational shearing.

**Figure 16:**
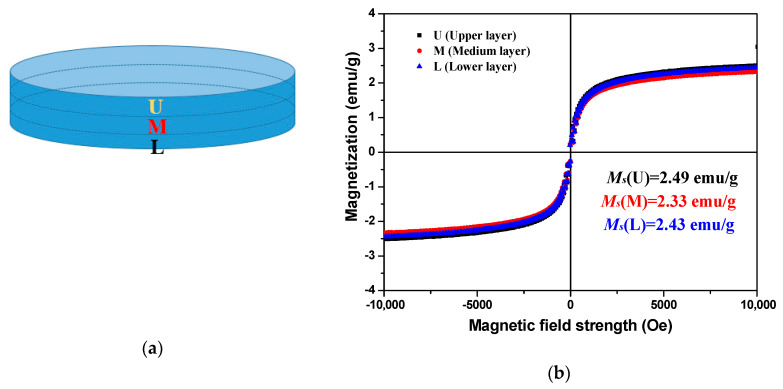
(**a**) Sample preparation and (**b**) magnetization curves of vibrating-sample magnetometer (VSM) measurement by vertically grinding into three layers.

Furthermore, the distribution of magnetic nanoparticles along the height of the paste disc was also examined. For this aim, a cementitious paste disc with thickness of 2 mm was prepared after the rheological tests under an external magnetic field of 0.4 T, which is the maximum reachable magnetic field strength of the used rheometer at a gap distance of 2 mm. The sample preparation process was similar to that of the samples with 1 mm. Once hardened, the paste disc was ground into three layers in the vertical direction, as shown in Figure 16a. The VSM experimental results of the corresponding ground powders are presented in Figure 16b. It can be seen that the saturation magnetization exhibited insignificant difference among the three vertical layers. This means that the nanoparticle migration in the vertical direction is not pronounced. In other words, the nanoparticle migration only occurs on the upper layer of the cementitious paste in the parallel plate. It can be concluded that the VSM results of different sections and layers are in good agreement with the proposed hypothesis.

## 5. Conclusions

In this study, the influence of an external magnetic field on the rheological properties of flowing cementitious paste containing nano-Fe_3_O_4_ particles was investigated. Distribution of the nanoparticles in the cementitious paste between parallel plates was analyzed by the magnetic properties of the powder as determined by VSM measurement. Based on the results and discussion, the main findings can be summarized as follows:

1. The addition of 3 wt.% nano-Fe_3_O_4_ particles significantly increases the measured shear stress, yield stress, and plastic viscosity due to the high water demand of the nanoparticles and the increase in inter-particle contacts. Under an external magnetic field of 0.5 T, the measured shear stress of the cementitious paste is lower than that obtained without a magnetic field, resulting in lower yield stress and higher plastic viscosity.
2. Obvious nanoparticle agglomeration and bleeding are observed on the interface between the cementitious paste and the upper rotating plate for the cementitious paste with nano-Fe_3_O_4_ particles but without a superplasticizer. The high magnetic field strength and high-rate shearing are two main influencing factors contributing to nanoparticle agglomeration.
3. High-rate shearing facilitates large magnetic cluster migration from the periphery of the plate to the inner sections due to the combined effect of the magnetic force gradient and shear rate gradient. For the center section of the plate, the coupled effects of magnetic force and high curvature lead to nanoparticles migrating away from this section. This migration behavior only occurs on the upper layer of the cementitious paste in the parallel plate of the rheometer. VSM measurements on the ground samples taken at different sections and layers provide a solid verification of the hypothesis regarding nanoparticle migration under the combined effect of magnetic field and high shearing.

## Figures and Tables

**Table 1:** Chemical composition of Portland cement [14].

Components	% By Mass
SiO_2_	19.6
Al_2_O_3_	4.88
Fe_2_O_3_	3.14
CaO	63.2
MgO	1.8
SO_3_	2.9
K_2_O	0.56
TiO_2_	0.25
ZnO	0.1
Others	3.57
